# Methadone-Induced Toxicity—An Unexpected Challenge for the Brain and Heart in ICU Settings: Case Report and Review of the Literature

**DOI:** 10.3390/life15071084

**Published:** 2025-07-10

**Authors:** Buzatu Georgiana Cristina, Sebastian Isac, Geani-Danut Teodorescu, Teodora Isac, Cristina Martac, Cristian Cobilinschi, Bogdan Pavel, Cristina Veronica Andreescu, Gabriela Droc

**Affiliations:** 1 Department of Anesthesiology and Intensive Care I, Faculty of Medicine, Carol Davila University of Medicine and Pharmacy, 020021 Bucharest, Romania; cristina.buzatu944@gmail.com (B.G.C.); geani-danut.teodorescu0721@stud.umfcd.ro (G.-D.T.); cristina.martac@drd.umfcd.ro (C.M.); gabriela.droc@umfcd.ro (G.D.); 2 Department of Internal Medicine II, Faculty of Medicine, Carol Davila University of Medicine and Pharmacy, 020021 Bucharest, Romania; teodora.isac@umfcd.ro; 3 Department of Anesthesiology and Intensive Care II, Carol Davila University of Medicine and Pharmacy, 050474 Bucharest, Romania; cristian.cobilinschi@umfcd.ro; 4 Department of Physiology, Faculty of Medicine, Carol Davila University of Medicine and Pharmacy, 020021 Bucharest, Romania; bogdan.pavel@umfcd.ro; 5 Department of Modern Languages, Faculty of Medicine, Carol Davila University of Medicine and Pharmacy, 020021 Bucharest, Romania; cristina.andreescu@umfcd.ro

**Keywords:** methadone, opioid substitution therapy, left ventricular failure, cardiogenic shock, brain insult, multidisciplinary care

## Abstract

Introduction: Methadone, a synthetic opioid used for opioid substitution therapy (OST), is typically associated with arrhythmias rather than direct myocardial depression. Neurological complications, especially with concurrent antipsychotic use, have also been reported. Acute left ventricular failure in young adults is uncommon and often linked to genetic or infectious causes. We present a rare case of reversible cardiogenic shock and cerebellar insult due to methadone toxicity. Case Presentation: A 37-year-old man with a history of drug abuse on OST with methadone (130 mg/day) was admitted to the ICU with hemodynamic instability, seizures, and focal neurological deficits. Diagnostic workup revealed low cardiac output syndrome and a right cerebellar insult, attributed to methadone toxicity. The patient received individualized catecholamine support. After 10 days in the ICU, he was transferred to a general ward for ongoing cardiac and neurological rehabilitation and discharged in stable condition seven days later. Conclusions: Methadone-induced reversible left ventricular failure, particularly when accompanied by cerebellar insult, is rare but potentially life-threatening. Early recognition and multidisciplinary management are essential for full recovery in such complex toxicological presentations.

## 1. Introduction

Acute left ventricular systolic dysfunction (LVSD) is a serious clinical condition characterized by impaired myocardial contractility, which leads to reduced cardiac output and potential organ hypoperfusion. Common causes include acute coronary syndromes, myocarditis, severe valvular diseases, stress-induced cardiomyopathy (Takotsubo), and toxic or drug-induced cardiomyopathies [1]. Among these, methadone, a synthetic opioid often used in opioid maintenance therapy, is rarely implicated in direct myocardial depression, with the primary concern being arrhythmogenic complications, such as QT interval prolongation and torsades de pointes [2].

Methadone-induced QT prolongation is a well-documented effect, primarily caused by the inhibition of hERG potassium channels, which play a crucial role in cardiac repolarization. This alteration increases the risk of arrhythmias, particularly torsades de pointes [2,3]. The emerging literature has begun to acknowledge the potential for methadone to exert direct myocardial toxic effects, including mitochondrial dysfunction and oxidative stress in cardiac myocytes [4]. However, case reports or series specifically describing isolated LV systolic dysfunction with echocardiographic recovery after methadone cessation remain sparse.

In addition to these direct effects, methadone toxicity can be further complicated by acute opioid withdrawal, which triggers a surge in catecholamines. This surge, in combination with methadone’s proarrhythmic properties, may induce stress-induced cardiomyopathy or worsen pre-existing left ventricular dysfunction [5,6]. Moreover, opioid-induced respiratory depression leads to hypoxia, exacerbating myocardial ischemia and impairing left ventricular function [7].

Diagnosing methadone-induced left ventricular systolic dysfunction can be difficult due to its subtle, nonspecific symptoms, which may resemble conditions like acute coronary syndrome, stress cardiomyopathy, or myocarditis. A detailed patient history, including recent methadone use or overdose, is crucial for narrowing the differential diagnosis. While ECG findings may show QT prolongation or other abnormalities, they are not always definitive for left ventricular dysfunction. Elevated serum biomarkers like troponins can be observed, but they may be influenced by opioid-related respiratory depression or hypotension [8].

Echocardiography often shows reduced left ventricular ejection fraction (LVEF), but additional assessment of pulmonary pressures and right ventricular function is needed to differentiate methadone-induced cardiotoxicity from other causes of acute heart failure, as methadone can exacerbate both left and right heart dysfunction [9].

Therapeutic management involves stabilizing hemodynamics using inotropic agents (dobutamine) and vasopressors (norepinephrine), correcting electrolyte imbalances (hypokalemia, hypomagnesemia, hypocalcemia), and ensuring continuous cardiac monitoring due to methadone’s long half-life. In severe cases, advanced circulatory support like VA-ECMO or IABP may be necessary [1,10].

Additionally, patients undergoing opioid substitution therapy (OST), particularly with methadone, and those receiving complex antipsychotic regimens such as quetiapine, may be at increased risk of ischemic cerebrovascular events. Case reports have described strokes temporally associated with both agents, raising concern about their potential contribution to neurovascular injury [11,12,13]. Methadone, a synthetic opioid with high affinity for μ-opioid receptors located in the cerebellum and basal ganglia, is known to induce neurotoxicity involving these regions, particularly in overdose scenarios. Moreover, the administration of quetiapine in low doses could also have an arrhythmic potential [14,15,16]. The concomitant use of quetiapine may further potentiate neurovascular vulnerability [17]. Notably, cerebellar infarctions, although uncommon, have been described in the setting of methadone toxicity, suggesting a possible predilection for posterior circulation involvement in such contexts [11].

This case report aims to highlight a rare association of acute left ventricular systolic dysfunction and cerebellar insult secondary to methadone toxicity, emphasizing the importance of early recognition, differential diagnosis, and appropriate multidisciplinary support in such cases.

## 2. Case Presentation

**Figure 1:**
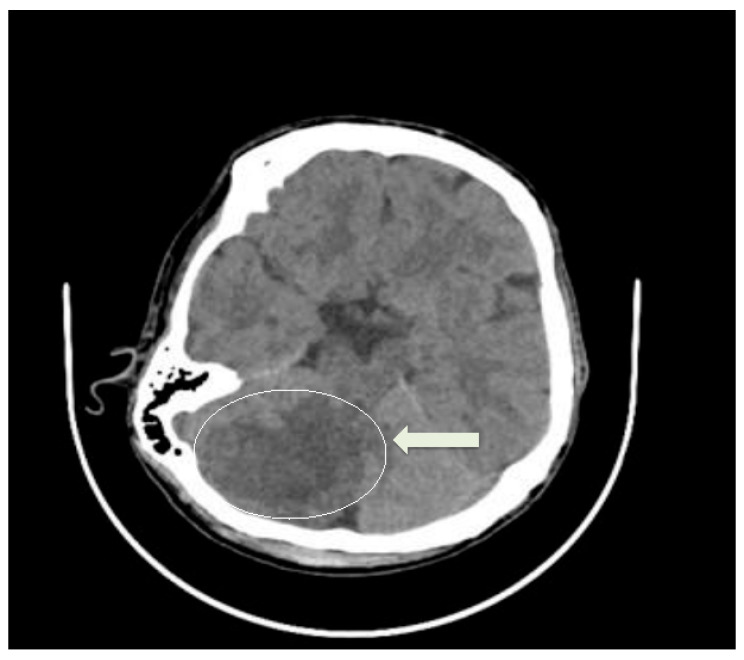
Brain CT showing right cerebellar insult (arrow).

A 37-year-old Caucasian male (70 kg, 170 cm) with a history of drug abuse under opioid substitution therapy (OST) with methadone (130 mg/day) had presented to the emergency department with episodes of syncope and convulsive seizures. Imaging studies confirmed a right cerebellar insult (Figure 1). Further, the patient was admitted to the intensive care unit (ICU) for hemodynamic instability and low cardiac output syndrome.

Past medical history revealed recent opioid detoxification, with negative toxicology screens for two months. He was undergoing pharmacologic management with actual doses of methadone (130 mg/day), alprazolam (1 mg/day), quetiapine (400 mg/day), escitalopram (10 mg/day), and gabapentin (900 mg/day), started two months ago and titrated to effect by a psychiatrist.

At ICU admission, the patient was conscious, partially oriented to self, with no signs of meningeal irritation. A neurological exam revealed slight right-sided motor weakness, dysarthria, and a right extensor plantar reflex. Respiratory examination showed spontaneous breathing under 4 L/min oxygen supplementation (SpO_2_ = 96%), with moderate respiratory effort and bibasilar crackles more pronounced on the left side. The arterial blood gas sample revealed hypokalemia, hypocalcemia, and lactic acidosis: pH = 7.37, pCO_2_ = 46 mmHg, pO_2_ = 88 mmHg, Na = 138 mmol/L, K = 3.06 mmol/L, Ca = 1.07 mmol/L, blood glucose = 208 mg/dL, lactate = 6.27 mmol/L, serum bicarbonate = 26 mmol/L, base excess = 0.9 mmol/L, hemoglobin = 13 g/dL. Blood tests revealed leukocytosis with neutrophilia: white blood cell count = 13.11 × 10^3^/μL; neutrophils = 11.30 × 10^3^/μL; CRP = 25.69 mg/L; presepsin = 262 pg/mL; procalcitonin = 0.37 ng/mL; a moderate hepatic cytolytic syndrome (AST = 316 U/L and ALT = 50 U/L), with normal renal function parameters (BUN = 30 mg/dL, creatinine = 0.79 mg/dL).

Hemodynamically, the patient was unstable: blood pressure = 90/55 mmHg, sinus tachycardia (HR = 105 bpm), capillary refill time > 3 s, and mottling of the lower limbs. Laboratory workup showed marked elevation of cardiac enzymes: CK: 29,494 U/L; CK-MB: 311.6 U/L; hs-cTnI: 10,528 ng/L; NT-proBNP: 25,396 pg/mL.

PiCCO monitoring revealed a cardiac index of 1.3 L/min/m^2^ and an elevated systemic vascular resistance index (SVRI) of 3200 dyn·s·cm^−5^·m^2^.

**Figure 2:**
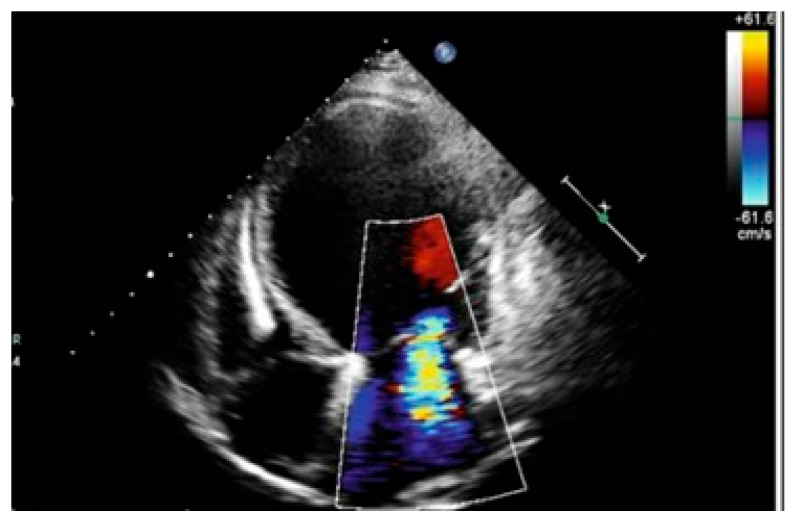
Echocardiographic findings (apical four-chamber view) at admission that revealed a grade III mitral regurgitation and mildly dilated left ventricle. The color scale reveals red tones for the blood approaching the transducer and blue tones for the blood moving away from the transducer.

The transthoracic echocardiography shows a mildly dilated left ventricle with severe systolic dysfunction due to diffuse hypokinesia (LVEF 20–25%), a dilated left atrium, a right ventricle with normal size but impaired function, grade III mitral regurgitation, and minimal tricuspid regurgitation (Figure 2).

**Figure 3:**
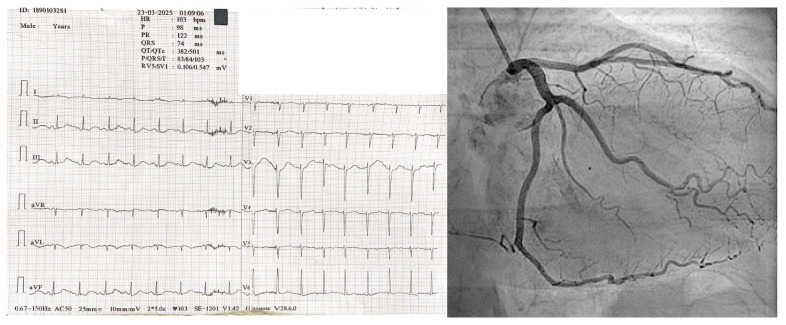
ECG tracing revealing prolonged QTc and signs of ischemia in the anterior limbs (aVL, V1–V4) (**left**) Coronary angiography showed no abnormalities (**right**).

Electrocardiogram revealed sinus rhythm and prolonged QTc (501 ms) (Figure 3), and coronary angiography showed patent coronary arteries (Figure 3).

**Figure 4:**
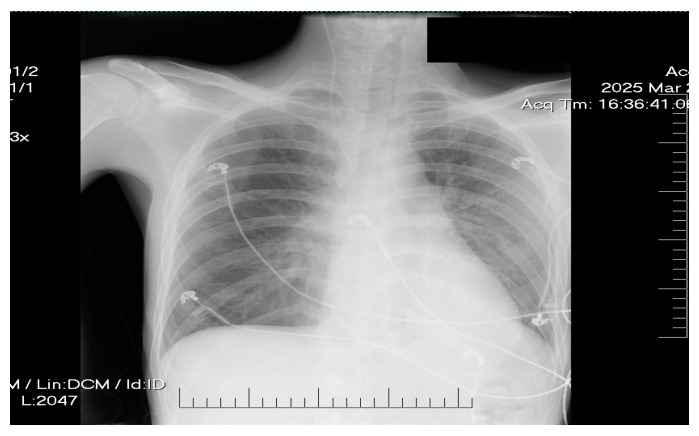
Chest X-ray revealing moderate pulmonary congestion.

Chest radiography showed mild pulmonary congestion (Figure 4).

The differential diagnosis included acute coronary syndrome (normal coronary angiography), myocarditis (absence of viral symptoms or typical imaging findings), Takotsubo cardiomyopathy (echocardiography showed diffuse rather than regional dysfunction), sepsis-induced cardiomyopathy (lack of a confirmed infection, negative markers for sepsis, only moderate inflammatory markers, and stable respiratory function), and neurogenic stunned myocardium (atypical damaged cerebral region—right cerebellum).

Based on the clinical presentation and imagistic and biochemical findings, we concluded that the patient suffered from cardiogenic shock secondary to methadone toxicity and associated right cerebellar insult.

Therapeutic management focused on both cardiologic and neurologic supportive strategies. Given the hemodynamic instability and evidence of low cardiac output, inotropic and vasopressor support was initiated. Dobutamine was administered with progressive titration, reaching a maximum dose of 9.52 μg/kg/min, to enhance the myocardial contractility. Simultaneously, norepinephrine was infused, up to 1.19 μg/kg/min, to maintain mean arterial pressure (MAP) above 65 mmHg and ensure adequate tissue perfusion. Diuretic therapy was initiated with Furosemide i.v. (20 mg t.i.d.) and spironolactone (50 mg/day). Electrolyte imbalances were promptly corrected with intravenous supplementation of potassium and calcium in the context of documented hypokalemia and hypocalcemia, both of which carry proarrhythmic potential, especially in the setting of QTc prolongation.

We added prophylactic therapy for secondary brain damage with mannitol 20% (0.25/kg/dose t.i.d.) and Acetylsalicylic acid (75 mg/day). The patient continued the previous treatment with alprazolam (1 mg/day), quetiapine (400 mg/day), escitalopram (10 mg/day), and gabapentin (900 mg/day). Given the context of methadone-induced left ventricular failure, we decided to reduce the methadone dose to 100 mg/day from the admission day until discharge in order to avoid exacerbation of opioid withdrawal-related catecholamine surges that could further destabilize the cardiovascular function.

**Figure 5:**
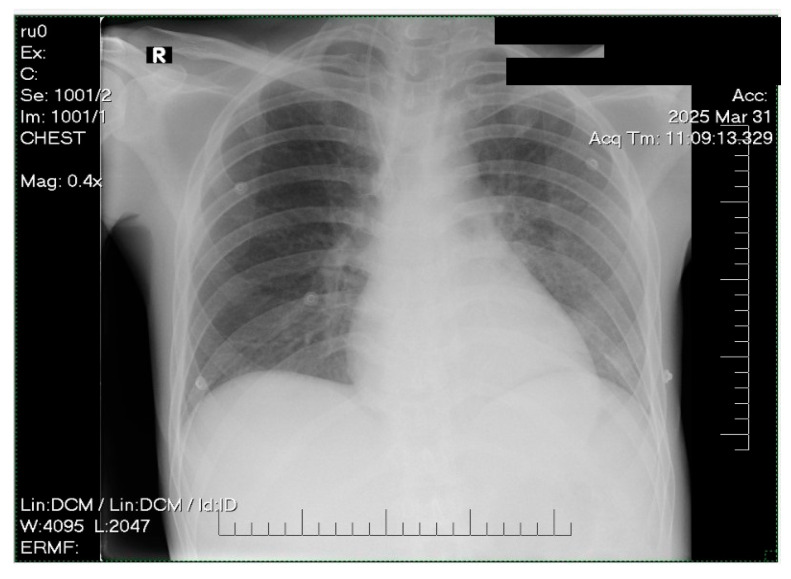
Chest X-ray showing no signs of pulmonary congestion or consolidation.

Supportive care included noninvasive ventilation (NIV) to manage moderate respiratory effort, with transition to supplemental oxygen as the clinical status improved. Empiric broad-spectrum antibiotic therapy with ceftriaxone (2 g/day) was initiated due to suspected left-sided basal bronchopneumonia identified on the chest radiograph, which was ceased on day 3 in the absence of the symptomatology and the improved chest X-ray (Figure 5).

Additional measures comprised stress ulcer prophylaxis with intravenous proton pump inhibitors (Pantoprazole 40 mg I.V./day) and venous thromboembolism prophylaxis with subcutaneous enoxaparin (40 mg/day). Glycemic control was ensured with i.v. rapid-onset insulin.

**Figure 6:**
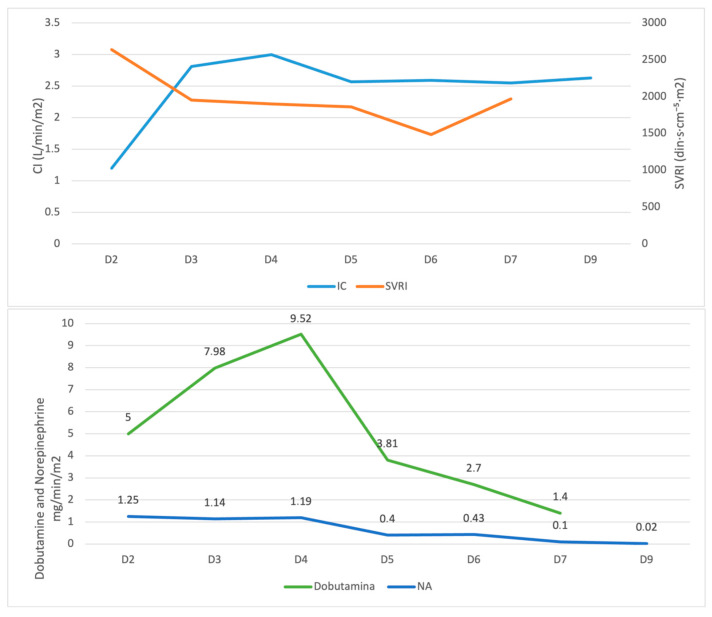
Hemodynamic evolution during vasopressor and inotrope weaning. Cardiac Index (CI, L/min/m^2^) and Systemic Vascular Resistance Index (SVRI, dyn·s·cm^−5^·m^2^) are shown on the upper panel. Dobutamine and norepinephrine doses (μg/kg/min) are shown on the lower panel. D represents days of ICU stay.

Over the following days, hemodynamics improved, enabling progressive tapering of both dobutamine and norepinephrine, followed by their complete discontinuation. Concurrently, the cardiac index increased and SVRI normalized, indicating improved cardiac output and vascular tone (Figure 6).

Between day 2 (D2) and day 9 (D9), CI gradually increased while SVRI decreased and stabilized. This improvement in the hemodynamic status allowed for progressive reduction and discontinuation of both vasopressors, reflecting enhanced myocardial function and vascular tone. Cardiac biomarkers progressively decreased, with CK-MB falling to 10.9 U/L, hs-cTnI to 112 ng/L, and NT-proBNP showing a steady decline to 10,072 pg/mL, reflecting ongoing myocardial recovery.

**Figure 7:**
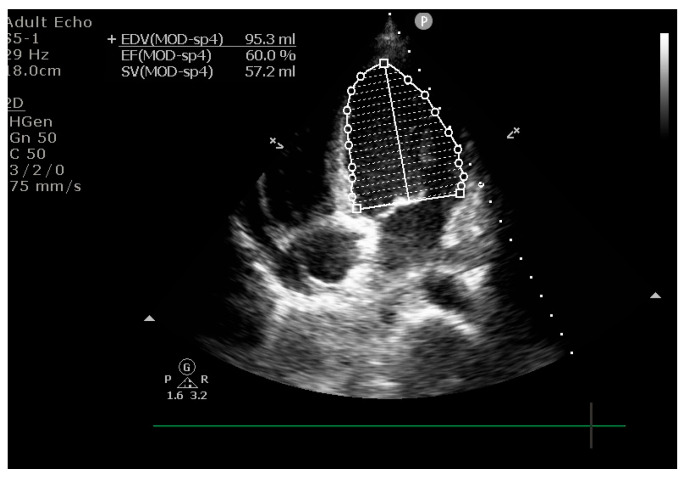
Echocardiographic findings showing an LVEF of 60%.

Echocardiography repeated on day 8 showed LVEF improvement to 60%, with reduced mitral regurgitation and normal longitudinal left ventricular function (Figure 7).

The patient was discharged from the ICU on day 10 and transferred for cardiac and neurological rehabilitation to the peripheric ward. Seven days later, the patient was discharged from the hospital with the recommendation to continue methadone substitution therapy at a dose of 100 mg/day, along with alprazolam (1 mg/day), quetiapine (400 mg/day), escitalopram (10 mg/day), and gabapentin (900 mg/day). The patient considered the ICU stay distressing but necessary in the context of cardiocirculatory support. The adherence to the prescribed interventions during the ICU stay was ensured through continuous monitoring and administration by the critical care team.

## 3. Discussion

This case illustrates a complex interaction between substance use, cardiovascular and neurologic compromises, and critical care management. Acute cardiac dysfunction in the context of methadone therapy and withdrawal highlights the complex cardiometabolic consequences of opioid use in critically ill patients [18]. Although methadone is typically associated with electrophysiological disturbances such as QT prolongation, recent insights suggest it may also exert direct negative inotropic effects, possibly via calcium channel interference or mitochondrial dysfunction, especially in high doses or in the context of withdrawal [19].

What sets this case apart is the presence of an isolated left ventricular systolic dysfunction (LVSD) with a prolonged QT interval, in the absence of the Takotsubo morphology [20]. Previous reports of methadone-associated cardiotoxicity have predominantly described right ventricular dysfunction, often related to acute pulmonary hypertension or stress-induced cardiomyopathy with transient apical ballooning [21,22]. To our knowledge, isolated non-Takotsubo LVSD in this context has rarely been documented. This suggests a possibly underrecognized presentation of methadone-induced cardiac dysfunction, potentially exacerbated by concurrent hypokalemia and hypocalcemia, both of which may also contribute to QT prolongation and impaired myocardial contractility. Individual variability in cardiac sensitivity to methadone or its metabolites could be possibly influenced by genetic polymorphisms affecting ion channels or drug metabolism.

In addition to the rare cardiovascular presentation, this case is further distinguished by the occurrence of a right cerebellar insult in the absence of conventional vascular risk factors. While a direct causal link cannot be established, the temporal association with both methadone and quetiapine therapy raises concern. Case reports have described cerebellar insult linked to methadone toxicity, with preferential involvement of the cerebellum and basal ganglia [16]. Quetiapine, independently, has been associated with increased stroke risk, even in younger patients without major cardiovascular comorbidities [12].

Notably, cerebellar infarctions are considered atypical in the context of generalized hypoperfusion and are more commonly associated with toxic-metabolic or drug-related mechanisms. In this context modern biochemical diagnostic tools for brain damage could be useful [23]. The posterior circulation localization in this case thus supports a hypothesis of direct neurotoxicity over systemic circulatory failure, reinforcing the importance of considering drug-induced etiologies in similar clinical contexts [16].

An important therapeutic principle demonstrated in this case is the decision to continue methadone during ICU care, despite its cardiotoxic potential. Abrupt cessation could have aggravated the patient’s instability via withdrawal-related autonomic dysregulation. This aligns with growing recognition that in patients undergoing opioid maintenance therapy, individualized continuation under close monitoring may be safer than sudden discontinuation in critical illness [18,24]. This strategy balances the risk of QT prolongation and arrhythmia with the dangers of autonomic dysregulation and undertreated withdrawal. While methadone maintenance treatment guidelines, including those from the Substance Abuse and Mental Health Services Administration and the World Health Organization, recommend individualized dosing based on symptom control and safety, they also emphasize caution in patients with cardiac risk [25,26].

In this case, methadone continuation likely contributed to the clinical stabilization, with supportive therapy directed at correcting electrolyte disturbances and optimizing cardiac function. This outcome supports a nuanced approach to methadone management, recognizing that its risks must be weighed against its role in maintaining neurovegetative stability in opioid-tolerant patients. Furthermore, this case contributes to a growing body of evidence that calls for greater vigilance and tailored protocols when managing opioid-maintained patients in critical care by highlighting the need for aggressive correction of modifiable risk factors such as electrolyte imbalances. Thus, this case provides valuable clinical insight into the potential mechanistically distinct myocardial toxicity of methadone by highlighting the importance of considering it as a potential etiology in patients with new-onset cardiomyopathy. To our knowledge, this presentation remains infrequently described in clinical case reports.

Regarding study limitations, we mention the lack of magnetic resonance imaging for a further differentiation of a toxic vs. ischemic cerebellar insult. Unfortunately, advanced neuroimaging could not be performed at admission due to patients’ instability. Furthermore, because of the clinical context and the good recovery process, we decided to postpone the MRI. Secondly, we did not focus the diagnostic strategy on any microvascular dysfunction of the heart, since the patient responded to supportive therapy and methadone dose adjustment accordantly.

## 4. Conclusions

In patients with a history of opioid dependence, particularly those undergoing substitution therapy, methadone should be recognized as a potential contributor to acute cardiac dysfunction and neurologic disturbance, including rare presentations such as cerebellar insult. Prompt identification, supportive care, and the continuation of maintenance therapy when appropriate are key to recovery. This case reinforces the need for awareness of drug-induced cardiac and neurological effects and the value of multidisciplinary care, including cardiac and neurological rehabilitation, for complete recovery.

## Data Availability

Data are contained within the article.
